# An in-depth analysis of postoperative insomnia in elderly patients and its implications on rehabilitation

**DOI:** 10.1007/s11325-024-03063-8

**Published:** 2024-06-11

**Authors:** Yuanqing Wang, Tianlong Wang, Shuai Feng, Ning Li, Yimeng Zhang, Yueyang Cheng, Hao Wu, Shuqin Zhan

**Affiliations:** 1 Department of Neurology, Xuanwu Hospital, Capital Medical University, 45 Changchun St. Xicheng District, Beijing, China; 2 Department of Neurology, People’s Hospital of Rizhao, Rizhao, China; 3 Department of Anesthesiology, Xuanwu Hospital, Capital Medical University, Beijing, China; 4 Department of Neurosurgery, Xuanwu Hospital, Capital Medical University, 45 Changchun St. Xicheng District, Beijing, China

**Keywords:** Sleep loss, Spine interbody fusion, Risk factor, Postoperative rehabilitation

## Abstract

**Objectives:**

(1) Assess the prevalence of postoperative insomnia; (2) identify the risk factors for postoperative insomnia before exposure to surgery; (3) explore the impact of postoperative insomnia on rehabilitation.

**Methods:**

A study was conducted with 132 participants aged ≥ 65 undergoing spine interbody fusion. We collected the basic demographic data, Numeric Rating Scales (NRS), Pittsburgh Sleep Quality Index (PSQI), Geriatric Depression Scale (GDS), and Beck Anxiety Inventory (BAI). We measured Quality of Recovery 40 (QoR-40), GDS, BAI, NRS, and PSQI on the first and third nights post-surgery, followed by QoR-40 and NRS assessments two weeks after surgery.

**Results:**

The cases of postoperative insomnia on the first and third nights and after two weeks were 81 (61.36%), 72 (54.55%), and 64 (48.48%), respectively, and the type of insomnia was not significantly different (*P* = 0.138). Sleep efficiency on the first night was 49.96% ± 23.51. On the first night of postoperative insomnia, 54 (66.67%) cases were depression or anxiety, and the PSQI was higher in this group than in the group without anxiety or depression (*P* < 0.001). PSQI, GDS, and the time of surgery were related factors for postoperative insomnia (*P*PSQI < 0.001,* P*GDS = 0.008, and *P*Time = 0.040). Postoperative rehabilitation showed differences between the insomnia and non-insomnia groups (*P* < 0.001).

**Conclusions:**

The prevalence of postoperative insomnia in the elderly was high, and postoperative insomnia had a significant correlation with postoperative rehabilitation. Interventions that target risk factors may reduce the prevalence of postoperative insomnia and warrant further research.

**Clinical Trial Registration:**

Multivariate analysis of postoperative insomnia in elderly patients with spinal surgery and its correlation with postoperative rehabilitation (https://www.chictr.org.cn/bin/project/edit?pid=170201; #ChiCTR2200059827).

**Supplementary Information:**

The online version contains supplementary material available at 10.1007/s11325-024-03063-8.

## Introduction

Insomnia, a prevalent global public health issue, is characterized by difficulties in sleep onset and sleep maintenance, early awakening, insufficient overall sleep duration, and/or impaired sleep quality. The elderly population is more susceptible to insomnia than other age demographics, especially those with underlying mental and physical disorders. Furthermore, the prevalence of insomnia has increased in older patients with a variety of mental and physical diseases. Over the last two decades, there has been a significant demographic shift towards an aging global population, thereby suggesting that the challenges of global geriatric care are significant issues that need timely addressal [1]. With advances in surgical and anesthetic methodologies and longer life expectancies, the number of elderly patients undergoing surgery has increased dramatically [2], accounting for 25–33.33% of all surgical patients [3]. Currently, more than half of all operations are performed on elderly patients in the United States, and this proportion will continue to increase [4]. Meanwhile, acute insomnia in perioperative adults can be as high as 79.1% [5], with even higher rates in the elderly.

Insomnia in the elderly affects daytime function and leads to deterioration of physical and mental health [6–9]. Perioperative insomnia is closely related to anxiety, depression, postoperative delirium, and decreased efficiency of wound healing, which further delays the postoperative rehabilitation process and increases the consumption of family and social resources. Therefore, strengthening the evaluation and management of perioperative insomnia has excellent clinical and social significance for improving the quality of life and prognosis in the elderly. This study had three objectives. First, we aimed to investigate the prevalence of insomnia in older patients following spine interbody fusion. Second, we explored potential factors such as age, sex, body mass index (BMI), insomnia history, education, underlying medical conditions, pain, frailty, anxiety, depression, and the time of surgery that can contribute to postoperative insomnia in this patient cohort to determine intervention targets. Third, we examined differences in the postoperative rehabilitation process, duration of hospitalization, and total hospital expenses between elderly patients with and without insomnia following spine interbody fusion. Although studies on postoperative insomnia have been conducted in the past, there is still a lack of research on postoperative insomnia and its correlation with postoperative rehabilitation in elderly patients undergoing spine interbody fusion. In particular, we screened out the risk factors for postoperative insomnia to provide a basis for developing intervention targets.

## Material and methods

### Participants

All non-emergent spine interbody fusions performed at Xuanwu Hospital, Capital Medical University from April 2022 to April 2023 were reviewed. Ethics approval for this study was obtained from the Human Research Ethics Committee (LYS-2022[051]) at 2022–04-20. A convenience sampling strategy was performed. Participants met the following inclusion criteria: ① Spine interbody fusion should be performed at an appropriate time; ② Age ≥ 65 years; and ③American Society of Anesthesiologists (ASA) physical status classification < IV including normal healthy patients and patients with mild or severe systemic disease whose function was in the early decompensation stage. The study excluded participants if they met at least one of the following criteria: ① A history of moderate-to-severe psychosis and/or psychoactive substance abuse; ② Presence of central nervous system disease; ③ Complications with severe organ insufficiency/failure; ④ Severe speech, vision, or hearing impairment, and/or dementia resulting in non-completion of the scale examination; ⑤ Non-24-h sleep rhythm screened with Morningness-Eveningness Questionnaire; and ⑥ Incompatibility with the clinical research protocols.

### Measurements and data collection

**Fig. 1:**
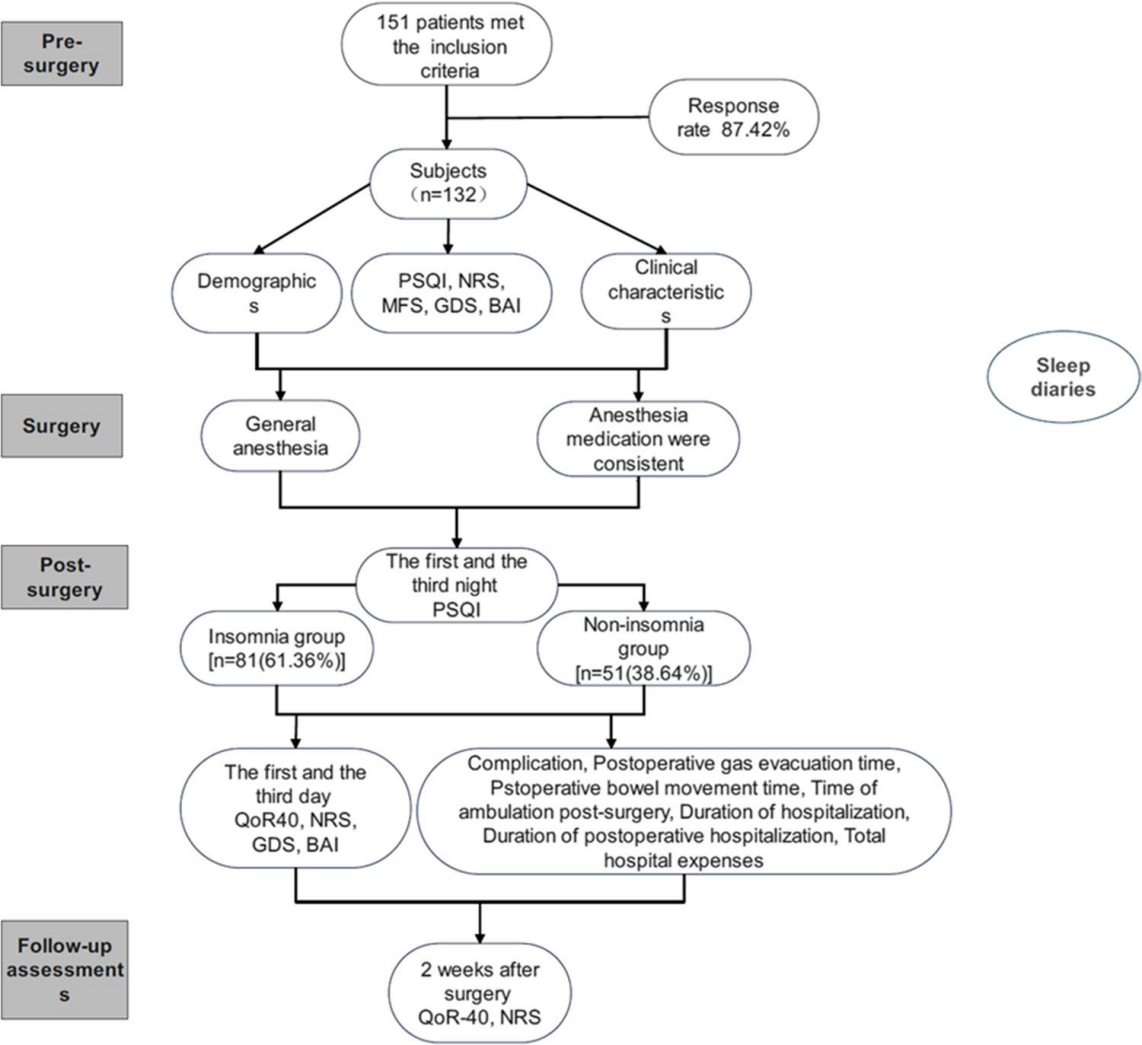
Flow diagram of the study**.** The entire procedure was categorized into presurgery, surgery, postsurgery stages, and a 2-week follow-up period. PSQI, Pittsburgh Sleep Quality Index; GDS, Geriatric Depression Scale; BAI, Beck Anxiety Inventory; MFS, Multidimensional Frailty Score; NRS, Numeric Rating Scale; QoR-40, Quality of Recovery 40

Professionally trained sleep physician with more than two years of work experience conducted a preoperative face-to-face structured interview to collect patient demographics and relevant clinical data including age, sex, BMI, insomnia history, education level, marital status, underlying medical conditions, Numeric Rating Scales (NRS), Multidimensional Frailty Score (MFS), Pittsburgh Sleep Quality Index (PSQI), Geriatric Depression Scale (GDS), and Beck Anxiety Inventory (BAI). On the first and third nights after surgery, the Quality of Recovery 40 (QoR-40) index, NRS, GDS, BAI, and PSQI were collected. Complications, postoperative gas evacuation time, and postoperative bowel movement time were documented. Moreover, the duration of hospitalization including the duration of postoperative hospitalization and total expenses in the hospital were recorded at discharge. Follow-up assessments using the NRS score and QoR-40 score were conducted 2 weeks after surgery through face-to-face or video structured interviews. Subjects were asked to maintain consensus sleep diaries pre-surgery and for 2 weeks post-surgery in the paper versions (Fig. 1). Insomnia diagnostic criteria were in accordance with the International Classification of Sleep Disorders-third Edition (ICSD-3).

### Pittsburgh sleep quality index

The PSQI has high reliability and effectiveness and is classified into seven components: subjective sleep quality, sleep latency, sleep duration, habitual sleep efficiency, sleep disturbances, use of sleeping medication, and daytime dysfunction.

### Quality of recovery 40

The QoR-40 was used to assess the quality of recovery. The QoR-40 has five dimensions of recovery after surgery and anesthesia: physical independence, patient support, comfort, emotions, and pain [10]. The QoR-40 has been validated in patients recovering from spine surgery [11].

### Multidimensional frailty score

The MFS has the concept of cumulative deficit and frailty, predicting adverse outcomes in patients undergoing intermediate to high-risk elective operations [12]. An MFS > 5 was classified as high-risk, showing increased postoperative mortality and longer postoperative hospital stays as their scores increased.

### Geriatric depression scale

The GDS has a higher rate of eligibility than other depression scales, and this advantage is more pronounced in older adults [13]. A total score of ≥ 11 points was considered indicative of depression.

### Beck anxiety inventory

The BAI is a self-report monitoring form and a brief measure of anxiety with a focus on somatic symptoms of anxiety that was developed as a measure adept at discriminating between anxiety and depression [14]. Anxiety was defined as a BAI score ≥ 45.

### Time of surgery

Cases were categorized based on "early start" or "late start," determined by return room time before or after 4:00 _PM_. General anesthesia was used. Anesthesia was induced with etomidate, sufentanil, and rocuronium bromide, and was maintained using propofol and remifentanil. Dexmedetomidine was used as a sedative and analgesic. There was a unified management of specialized anesthesiologists during the operation.

### Statistical analysis

The descriptive statistical method included the mean ± standard deviation, composition ratio, as well as the median and quartile. When data conformed to normal distribution, we used an unpaired, two-tailed *t*-test to analyze continuous variables, while using the Wilcoxon signed-rank test to analyze non-normally distributed continuous variables. For categorical variables, we used a Pearson chi-square test for analysis. Binary logistic regression was used to perform correlation analysis of variables. The SPSS 26.0 software (IBM Corporation, Armonk, NY, USA) was used for statistical analysis of all data. Statistical significance was defined as *P* < 0.05.

## Results

### Demographics and clinical features

A total of 151 cases of postoperative patients with spine interbody fusion were initially screened, with a response rate of 87.42%. Finally, 132 cases were included. The preoperative and first postoperative day interviews were conducted through face-to-face structured visits. On the third postoperative day, 127 patients underwent face-to-face interviews, while five patients had structured video interviews. Furthermore, 110 patients had follow-up assessments 2 weeks after surgery through face-to-face structured interviews, while 32 patients had structured video interviews. The mean age of the included patients was 71 years (range: 65–88 years). The PSQI of all subjects was 8.00 (6.00, 12.00), and the sleep efficiency was 76.16% ± 14.48. There were 77 (58.33%) patients with insomnia and 55 (41.67%) subjects without insomnia before surgery. There were 10 patients with new acute insomnia before surgery, and the incidence of acute insomnia before surgery was 18.18%. The demographic and clinical characteristics are presented in Table 1.

**Table 1:** Demographic and Clinical Characteristics: Combined Insomnia and Non-insomnia Groups (n = 132)

Variables	Insomnia	Non-insomnia	Test Statistic	*P*
	n = 81 (61.36%)	n = 51 (38.64%)		
Sex male, n (%)	26 (32.10%)	27 (52.94%)	*χ*^*2*^ = 5.657	0.017^a^
Age, years	72.00 (67.50–78.00)	70.00 (67.00–73.00)	Z = − 1.721	0.085^b^
BMI (kg/m^2^)	24.99 ± 4.01	24.56 ± 2.92	*t* = − 0.712	0.478^c^
Higher education, n (%)	58 (71.60%)	42 (82.35%)	*χ*^*2*^ = 1.968	0.161^a^
Marriage, n (%)	76 (93.83%)	48 (94.12%)	*χ*^*2*^ = 0.000	1.000^a^
Underlying medical conditions, n (%)	66 (81.48%)	37 (72.55%)	*χ*^*2*^ = 1.457	0.227^a^
PSQI (pre)	10.81 ± 3.45	5.55 ± 2.54	*t* = − 10.075	< 0.001^c^
Sleep efficiency (pre)	72.22 (62.02–83.33) %	85.71 (76.47–91.67%)	Z = − 4.394	< 0.001^b^
SOI and SMI, n (pre %)	46 (57.79%)	3 (5.88%)	*χ*^*2*^ = 5.615	0.06^a^
GDS (pre) score	19.00 (7.00–27.00)	2.00 (1.00–4.00)	Z = − 7.988	< 0.001^b^
BAI (pre) score	42.00 (32.00–51.50)	28.00 (27.00–32.00)	Z = − 7.158	< 0.001^b^
MFS	4.00 (2.00–6.00)	2.00 (1.00–3.00)	Z = − 5.255	< 0.001^b^
NRS (pre)	6.00 (3.00–8.00)	1.00 (1.00–5.00)	Z = − 4.855	< 0.001^b^
Early start, n (%)	44 (54.32%)	34 (66.67%)	*χ*^*2*^ = 1.973	0.160^a^

Results are expressed as mean ± standard deviation if not otherwise stated, and in brackets as median (IQR). Higher education, the level of education is higher than K-12 education; Underlying medical conditions, diseases except for spinal disease; Early start: return room time before 4:00 _PM_.; sleep efficiency, the proportion of sleep relative to the time between lights out and final awakening. ^a^ Chi-square test;^b^ Mann–Whitney *U* test; ^c^ Independent-samples *t*-test

BMI, Body Mass Index, kg/m^2^; PSQI, Pittsburgh Sleep Quality Index; GDS, Geriatric Depression Scale; BAI, Beck Anxiety Inventory; MFS, Multidimensional Frailty Score; NRS, Numeric Rating Scale; pre, preoperative

### Postoperative insomnia

**Fig. 2:**
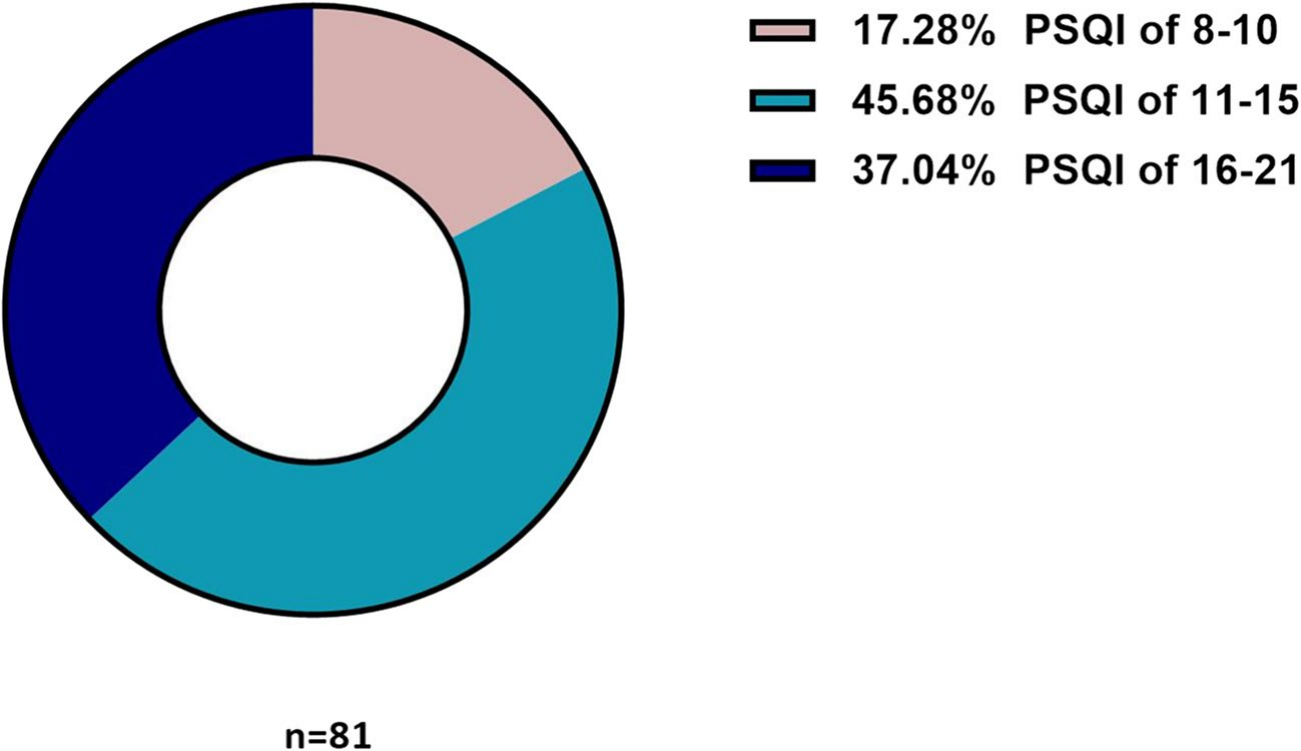
PSQI distribution of the insomnia group**.** On the first night after surgery, 81 patients experienced postoperative insomnia. Among them, 17.28% had a PSQI score ranging from 8 to 10, while 45.68% had scores between 11 and 15. The remaining 37.04% had scores ranging from 16 to 21. PSQI, Pittsburgh Sleep Quality Index

**Fig. 3:**
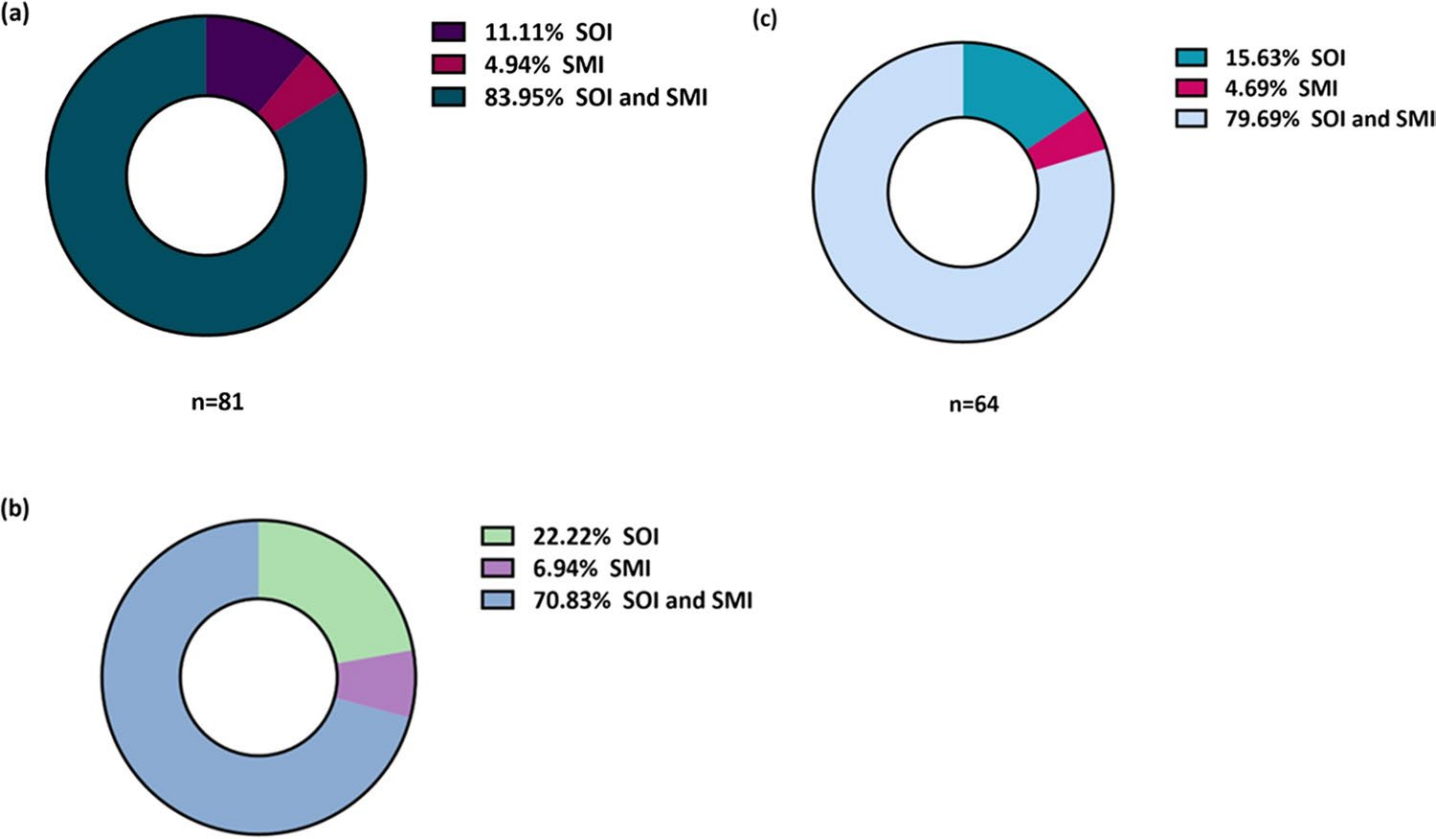
The type of postoperative insomnia over time**.** There was no difference in the type of insomnia during the three nights after surgery. **a** The type of insomnia on the first night after surgery, **b** The type of insomnia on the third night after surgery, **c** The type of insomnia 2 weeks after surgery. SOI, sleep onset insomnia; SMI, sleep maintenance insomnia

In our study, 61.36% patients (n = 81, 26 male and 55 female) were diagnosed with postoperative insomnia on the first night of the surgery. The details of PSQI on the first night after surgery are presented in Fig. 2. The average score of PSQI on the first night after surgery was 15.00 (interquartile range [IQR] 12.00–16.50), and the sleep efficiency was 49.96% ± 23.51. Among them, 9 patients (1 male and 8 female) had sleep onset insomnia (SOI), 4 patients (1 male and 3 female) had sleep maintenance insomnia (SMI), and 68 patients (24 male and 44 female) had both SOI and SMI. The details of the types of postoperative insomnia on the first night are presented in Table 2. On the third night after surgery, 72 patients (54.55%, 23 male and 49 female) had postoperative insomnia, and the sleep efficiency was 69.79% ± 16.20. In addition, after 2 weeks, 64 patients (48.48%, 18 male and 46 female) had postoperative insomnia, and the sleep efficiency was 72.29% ± 13.30. There was no statistically significant difference in the type of insomnia on the postoperative first and third nights and at 2 weeks (*P* = 0.138, Fig. 3). Before surgery, 77 patients had insomnia, and the prevalence of postoperative insomnia was 87.01%. There were 55 patients without insomnia before surgery, and the prevalence of postoperative insomnia was 25.45%. There was a statistically significant difference between the two groups (*P* < 0.001). Data on the prevalence of postoperative insomnia between patients in the preoperative insomnia group and the non-insomnia group is provided in Supplementary Table S1. According to the sleep diary, 8 patients showed no change or improvement in sleep after surgery; whereas, 124 patients experienced a decrease in postoperative sleep quality. Consistent with this, the average sleep resumption time was 3 (IQR 2.00–4.75) days.

**Table 2:** Characteristics of the First Night Postoperative Insomnia (n = 81)

Type	SOI or SMI	SOI and SMI	Test Statistic	*P*
	n = 13 (16.049%)	n = 68 (83.951%)		
Sex male, n (%)	2 (15.38%)	24 (35.29%)	*χ*^*2*^ = 1.176	0.278^a^
Age	70.00 (65.00–77.00)	73.00 (68.00–78.00)	Z = − 1.328	0.184^b^
PSQI	12.00 (9.50–15.00)	15.00 (12.00–17.00)	Z = − 2.034	0.042^b^
Sleep efficiency (%)	81.24 ± 15.41	43.98 ± 19.78	*t* = 6.419	< 0.001^c^

Results are expressed as mean ± standard deviation or median (IQR). sleep efficiency, the proportion of sleep relative to the time between lights out and final awakening. ^a^ Chi-square test; ^b^ Mann–Whitney *U* test; ^c^ Independent-samples *t*-test

PSQI, Pittsburgh Sleep Quality Index; SOI, sleep onset insomnia; SMI, sleep maintenance insomnia

On the first night of postoperative insomnia, 54 patients (66.67%, 16 male and 38 female) were diagnosed with depression or anxiety. Meanwhile, 27 patients (33.33%, 10 male and 17 female) had insomnia without anxiety or depression. There were statistically significant differences in anxiety and depression scores and PSQI between the two groups (*P* < 0.001), but no statistically significant differences between the two groups in terms of sex (*P* = 0.501) (Table 3). PSQI and sleep efficiency on the first day after surgery showed no statistically significant differences between male and female patients (*P* > 0.05) (Supplementary Table S2).

**Table 3:** First Postoperative Night Insomnia with/without Anxiety or Depression (n = 81)

Type	Insomnia with Anxiety or Depression	Insomnia without Anxiety or Depression	Test Statistic	*P*
	n = 54 (66.67%)	n = 27 (33.33%)		
PSQI	15.50 (13.00–17.00)	12.00 (9.00–14.00)	Z = − 4.372	< 0.001^a^
GDS score	26.00 (18.00–29.00)	4.00 (1.00–7.00)	Z = − 7.318	< 0.001^a^
BAI score	50.83 ± 13.62	30.00 ± 4.47	*t* = − 10.194	< 0.001^b^
Sex male, n (%)	16 (29.63%)	10 (37.04%)	*χ*^*2*^ = 0.453	0.501^c^

Results are expressed as mean ± standard deviation or median (IQR). ^a^ Mann–Whitney *U* test; ^b^ Independent-samples *t*-test; ^c^ Chi-square test

PSQI, Pittsburgh Sleep Quality Index; GDS, Geriatric Depression Scale; BAI, Beck Anxiety Inventory

### Risk factors for postoperative insomnia before exposure to surgery

**Fig. 4:**
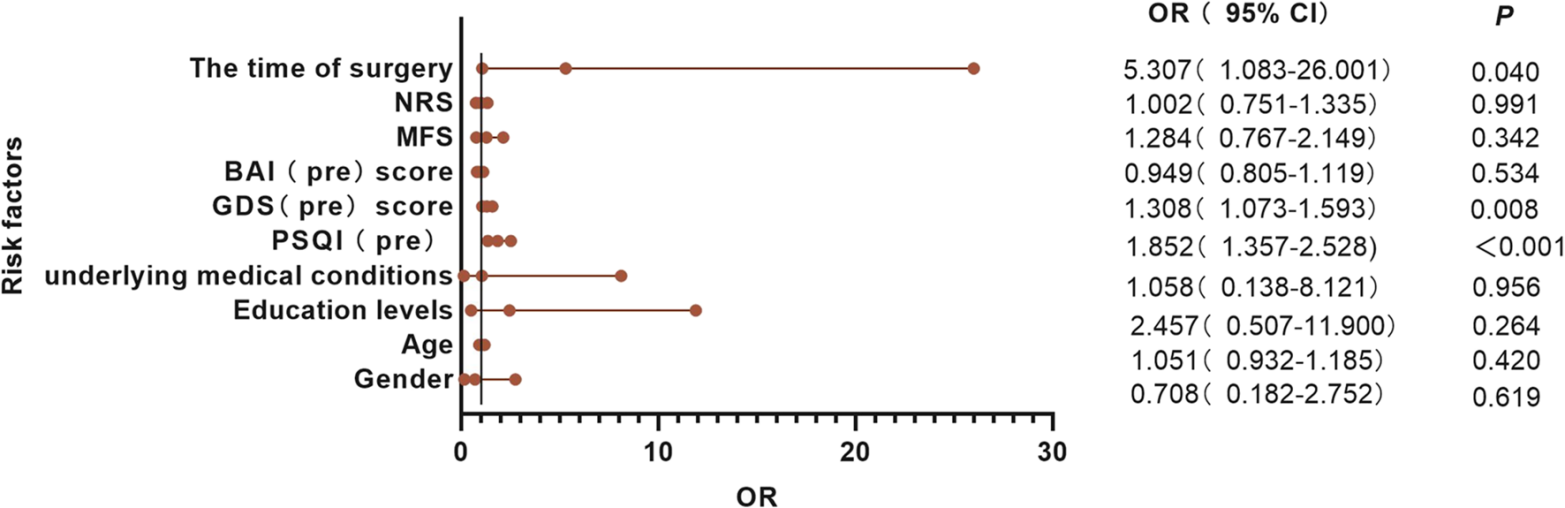
Preoperative risk factors for postoperative insomnia. PSQI (pre), GDS (pre), and the time of surgery were related factors for postoperative insomnia (*P-value* for PSQI < 0.001, *P-value* for GDS = 0.008, *P-value* for Time = 0.040). The time of surgery, determined by return room time before or after 4:00 _PM_ divided into "early start" or "late start"; Education level, divided into "higher than K-12 education" or "K-12 education and below"; Underlying medical conditions, diseases except for spinal disease. PSQI, Pittsburgh Sleep Quality Index; GDS, Geriatric Depression Scale; BAI, Beck Anxiety Inventory; MFS, Multidimensional Frailty Score; NRS, Numeric Rating Scale; pre, preoperative; OR, odds ratio; CI, confidence interval

A binary unconditional logistic regression model was established, and the forward method was used to select and eliminate independent variables. The regression analysis showed that the PSQI (pre), GDS (pre) score, and time of surgery were factors significantly related to postoperative insomnia (*P-value* for PSQI: < 0.001, *P-value* for GDS: 0.008, *P-value* for Time: 0.040). Analyses revealed that patients undergoing late-start surgery were 5.307 times more likely to experience postoperative insomnia than those undergoing early-start surgery (95% confidence interval [CI] = 1.083–26.001), making it the most significant associated factor (Fig. 4).

### Correlation of postoperative insomnia with postoperative rehabilitation

Of the 132 patients, 81 (61.36%) experienced postoperative insomnia, and 51 (38.64%) patients experienced no postoperative insomnia on the first night after surgery. There were significant differences in QoR-40 score and NRS scores between the two groups (*P* < 0.001). Furthermore, there were significant differences between the insomnia and non-insomnia groups in terms of postoperative gas evacuation time, postoperative bowel movement time, and time of ambulation post-surgery (*P* < 0.001). Conversely, there were no statistically significant differences between the two groups in terms of duration of hospitalization, duration of postoperative hospitalization, total hospital expenses, and complications (*P* > 0.05) (Table 4).

**Table 4:** Postoperative Insomnia’s Correlation with Postoperative Rehabilitation

Measure	Insomnia	Non-insomnia	Test Statistic	*P*
	n = 81 (61.36%)	n = 51 (38.64%)		
QoR-40 score				
First postoperative day	180.00 (175.50–188.00)	195.00 (190.00–197.00)	Z = − 6.925	< 0.001^a^
Third postoperative day	188.00 (183.00–193.00)	198.00 (195.00–199.00)	Z = − 7.215	< 0.001^a^
2 weeks postoperatively	197.00 (193.00–199.00)	200.00 (200.00–200.00)	Z = − 5.862	< 0.001^a^
NRS score				
First postoperative day	6.00 (3.00–7.00)	1.00 (0.00–3.00)	Z = − 5.905	< 0.001^a^
Third postoperative day	3.00 (2.00–6.00)	0.00 (0.00–2.00)	Z = − 6.205	< 0.001^a^
2 weeks postoperatively	1.00 (0.00–2.00)	0.00 (0.00–0.00)	Z = − 5.629	< 0.001^a^
Complication, n (%)	3 (3.70%)	0	*χ*^*2*^ = 0.625	0.429^b^
Postoperative gas evacuation time	24.00 (6.00–31.00)	3.00 (2.00–7.00)	Z = − 5.433	< 0.001^a^
Postoperative bowel movement time	3.00 (2.00–4.00)	1.00 (1.00–3.00)	Z = − 5.468	< 0.001^a^
Time of ambulation post-surgery	2.00 (2.00–3.00)	1.00 (1.00–2.00)	Z = − 4.803	< 0.001^a^
Duration of hospitalization	9.00 (8.00–11.00)	9.00 (8.00–11.00)	Z = − 0.895	0.371^a^
Duration of postoperative hospitalization	5.00 (4.00–7.00)	5.00 (4.00–6.00)	Z = − 0.901	0.368^a^
Total hospital expenses ($)	15,902.22 (14,001.24–24,826.10)	19,098.98 (13,121.70–25,004.28)	Z = − 0.381	0.703^a^

Results are expressed as median (IQR). ^a^ Mann–Whitney *U* test; ^b^ Chi-square test

QoR-40, Quality of Recovery 40; NRS, Numeric Rating Scale

## Discussion

To our knowledge, postoperative insomnia in elderly patients with spine interbody fusion has not yet been investigated. This study is to examine the prevalence, severity, and risk factors associated with postoperative insomnia in elderly patients undergoing spine interbody fusion and its implications on rehabilitation. Our findings revealed that over 61% of patients were diagnosed with postoperative insomnia on the first night after surgery, with sleep efficiency being less than 50%. There was no significant difference in the type of insomnia over time. In a study of elderly patients aged ≥ 65 years undergoing joint replacement, the prevalence of perioperative insomnia was 88.1%, with 57% experiencing sleep disruption [15]. In another study, polysomnography (PSG) was used to demonstrate changes in sleep patterns of patients with obstructive sleep apnea in the first week after surgery. The total sleep time on the first night after surgery reduced, and deep sleep time and rapid eye movement (REM) sleep also significantly reduced. Sleep efficiency did not return to normal until the seventh night after surgery [16], and these objective records were consistent with the subjective description of the patients. While in our study, the average sleep resumption time was 3 days. Another study found that REM sleep was significantly reduced on the first night after surgery and would return to preoperative baseline on the third night after surgery [17], consistent with our conclusions. Different findings may correlate with different types of surgery.

In this study, the incidence of acute insomnia before surgery was 18.18%. Wesselius et al. found that sleep efficiency was lower in the hospital than at home [18]. Consistent with our conclusion, this may be related to changes in the sleep environment, concerns about surgery, and other relevant factors. The incidence of postoperative insomnia was 25.45%, lower than expected. It may likely be because that a large proportion of patients had pain from spinal disease before surgery, which can affect sleep, and pain relief after surgery. However, the incidence and prevalence of postoperative insomnia in elderly patients undergoing interbody fusion have not been reported. More data from future research studies based on the current findings will be very valuable.In our study, the PSQI (pre), GDS (pre) score, and time of surgery were significant contributing factors for postoperative insomnia which many seem modifiable. A study on postoperative gastric cancer patients found that factors such as age, sex, and cancer stage were not associated with the degree of insomnia [19], while Ohayon believed that age and gender were associated with insomnia in cancer patients [20]. It is possible that different assessment tools and analytical methods might lead to different conclusions. In our study, the time of surgery was a related factor for postoperative insomnia. In another study, Song found that a greater degree of subsequent sleep disruption may occur when surgery was performed at night (18:00–22:00) than during the day (08:00–12:00). Additional, complication rates were significantly higher when surgery started in the afternoon or at night [21]. Ricci et al. compared daytime (06:00–16:00) to after-hours (16:00–06:00) surgery and found that after-hour surgeries were predictors for higher general complication rates, reoperation [22]. Similarly, Kenan found that starting surgery on non-emergent cardiac cases later in the day was associated with a two-fold absolute and risk-adjusted mortality [23]. Identifying patients at risk and providing preventive treatments tailored to risk factors before surgery has been suggested as a potential method to reduce the prevalence of postoperative insomnia in elderly patients [24]. In our observational cohort study, the risk factors for postoperative insomnia were identified through multivariate logistics regression analysis.

We found that the PSQI, anxiety, and depression scores on the first night after surgery were significantly higher in the insomnia group than in the non-insomnia group. Consistent with our findings, Hoang showed that the prevalence and severity of insomnia in cancer patients were related to the emotional score of the participants [25]. Female patients tended to be more anxious than male patients before surgery [26–28]; this was inconsistent with our results. Possibly, different surgeries lead to different conclusions. Preoperative anxiety has a high prevalence, ranging from 11 to 80% across different clinical disciplines [29]. Individuals with insomnia displayed more anxiety symptoms [30], while individuals with anxiety were more likely to experience insomnia [31]. Insomnia and anxiety reportedly co-exist in a vicious circle owing to a variety of mechanisms such as metabolism, immune function, and neurogenesis [32]. However, to our knowledge, most of these interventions targeting mood and insomnia have never been tested in general wards; therefore, prospective interventional studies are needed.

This study has some limitations. First, we conducted a basic analysis of the correlation between postoperative insomnia and factors such as age, sex, education levels, PSQI, MFS, BAI, GDS, and NRS. Other factors may also play a role but were not explored. Second, the insomnia of the included patients in this study was judged based on the evaluation of PSQI, but it might be wrong judgment based on patients’ recollection error. Third, based on the results of this study, it is valuable to set up a control group for further analysis.

## Conclusion

With the sharp increase in global aging and the rapid development of sleep medicine, the study of postoperative insomnia in the elderly is very relevant. Our results highlight a high prevalence of postoperative insomnia in elderly patients undergoing spine interbody fusion, with a significant correlation observed between postoperative insomnia and rehabilitation outcomes. Identifying modifiable factors can inform targeted interventions to enhance postoperative recovery. This study opens up new possibilities for perioperative management strategies for elderly patients undergoing spine interbody fusion, offering valuable theoretical support for post-surgical recovery enhancement.

## Supplementary Information

Below is the link to the electronic supplementary material.

Supplementary file1 (DOCX 19 KB)

## Data Availability

The data that support the study of this study are available from the corresponding author, [SZ and HW], upon reasonable request.
