# Supplementary material for: An in-depth analysis of postoperative insomnia in elderly patients and its implications on rehabilitation
Source: Sleep Breath. 2024 Jun 11;28(5):2187–95. doi: 10.1007/s11325-024-03063-8 (PMC11450051; doi:10.1007/s11325-024-03063-8)
Supplement: Supplementary file 1 — Supplementary file1 (DOCX 19 KB) [file 11325_2024_3063_MOESM1_ESM.docx]

**Table S1** Prevalence of Postoperative Insomnia between Patients in the Preoperative Insomnia Group and the Non-insomnia Group

| Measure | Preoperative Insomnia Group | Preoperative non-insomnia Group | Test Statistic | *P* |
| --- | --- | --- | --- | --- |
| Prevalence of insomnia on the first postoperative day | 87.01% | 25.45% | *χ^2^* = 51.280 | < 0.001^a^ |
| Prevalence of insomnia on the third postoperative day | 84.42% | 12.73% | *χ^2^* = 66.503 | < 0.001^a^ |
| Prevalence of insomnia at 2 weeks postoperatively | 77.92% | 7.27% | *χ^2^* = 64.114 | < 0.001^a^ |

^a^ Chi-square test

**Table S2** Sleep Based on Patients’ Sex on the First Day after Surgery (n = 81)

| Measure | Male | Female | Test Statistic | *P* |
| --- | --- | --- | --- | --- |
|  | n = 26 (32.10%) | n = 55 (67.90%) |  |  |
| PSQI | 9.83 ± 5.12 | 11.3 ± 4.74 | *t* = 1.695 | 0.092^a^ |
| Sleep efficiency | 63.91% ± 22.48 | 57.61% ± 26.41 | *t* = -1.423 | 0.157^a^ |

Results are expressed as mean ± standard deviation. ^a^ Independent-samples *t*-test. Sleep efficiency, the proportion of sleep relative to the time between lights out and final awakening.

PSQI, Pittsburgh Sleep Quality Index
